# MITF Regulates Downstream Genes in Response to *Vibrio parahaemolyticus* Infection in the Clam *Meretrix Petechialis*

**DOI:** 10.3389/fimmu.2019.01547

**Published:** 2019-07-04

**Authors:** Shujing Zhang, Xin Yue, Jiajia Yu, Hongxia Wang, Baozhong Liu

**Affiliations:** ^1^CAS Key Laboratory of Experimental Marine Biology, Center for Ocean Mega-Science, Institute of Oceanology, Chinese Academy of Sciences, Qingdao, China; ^2^University of Chinese Academy of Sciences, Beijing, China; ^3^Laboratory for Marine Biology and Biotechnology, Qingdao National Laboratory for Marine Science and Technology, Qingdao, China

**Keywords:** MITF, signaling pathway, *Vibrio parahaemolyticus*, clam, immune response

## Abstract

The microphthalmia-associated transcription factor (MITF) is a basic helix-loop-helix-leucine zipper protein that plays a key role in cell proliferation, survival and immune defense through the direct transcriptional control of downstream genes. We have found that MITF participates in the immune response to *Vibrio parahaemolyticus* infection in the clam *Meretrix petechialis*. In this study, we focused on how MITF functions in immunity. First, *PO, CTSK*, and *BCL-2* were identified as the target genes of MpMITF in the clam by RNAi. EMSAs showed direct binding between the MpMITF protein and the E-box of the *MpPO, MpCTSK*, and *MpBCL-2* promoters. Yeast one-hybrid assays also suggested that MpMITF could activate the expression of these three downstream genes. These results demonstrated that the transcriptional expression of *MpPO, MpCTSK*, and *MpBCL-2* is directly regulated by MpMITF. Second, we analyzed the roles of *MpPO, MpCTSK*, and *MpBCL-2* in clam immunity. The mRNA expression of *MpPO, MpCTSK*, and *MpBCL-2* increased significantly after *V. parahaemolyticus* challenge, which implied that these genes might take part in the immune defense against *V. parahaemolyticus* challenge in clams. The purified recombinant proteins, MpPO and MpCTSK, inhibited the growth of *V. parahaemolyticus*. Additionally, the apoptosis rate of clam haemocytes rose significantly when the activity of *MpBCL-2* was suppressed. These results revealed that *MpPO, MpCTSK*, and *MpBCL-2* are involved in the immune defense against *V. parahaemolyticus*. This study supports the idea that the MpMITF pathway plays a key role in immune defense through the direct regulation of the downstream genes *MpPO, MpCTSK*, and *MpBCL-2* in the clam, *M. petechialis*.

## Introduction

The microphthalmia-associated transcription factor (MITF), a member of the MYC family of transcription factors, is a basic helix-loop-helix-leucine zipper (bHLH-LZ) protein (1–4). MITF directly regulates downstream genes depending on its bHLH-LZ domain, which binds to the canonical E-box sequence (CANNTG) found in the promoter regions of downstream genes (5). MITF has been found in vertebrate species including mouse, rat, chicken, hamster, and human (6). In addition, MITF has been described in some invertebrates, such as *Caenorhabditis elegans, Drosophila melanogaster*, and *Halocynthia roretzi* (7). To date, MITF has been reported to be expressed mainly in melanocytes, osteoclasts, macrophages, B cells and natural killer cells and is the key regulator of signaling pathways that control cell proliferation, survival and immune defense (8).

MITF transcriptionally regulates a number of target genes involved in the immune signaling pathways of vertebrates, such as *phenoloxidase* (*PO*), *cathepsin K* (*CTSK*), *cyclin-dependent kinase 2* (*CDK2*), *N*-deacetylase/*N*-sulfotransferase (*NDST), perforin, tryptase*, and the anti-apoptotic factor *BCL-2* (9–15). Among them, *PO* is the key enzyme in the process of melanin production, which plays a role in immune defenses, wound healing and self/non-self-recognition mechanisms (16, 17). Moreover, as a member of the cysteine protease papain superfamily, the upregulation of *CTSK* is associated with various pathological processes in humans, such as inflammatory diseases and cancer (18). BCL-2 is an integral intracellular membrane protein, which is a critical regulator of mitochondrial membrane permeability and of the apoptotic mitochondrial pathway (19). In addition, MITF has been reported to regulate the target gene *tryptase* in the differentiation and functioning of mast cells (20), and mutated MITF can suppress the transactivation of *perforin* to reduce the natural killer activity of NK cells (13). All of these studies have demonstrated that MITF is a transcriptional regulator of innate immune signaling pathways in vertebrates, but such mechanisms in invertebrates remain poorly understood.

The clam *Meretrix petechialis* is a commercial mollusc found off the coast of southern and southeast Asia (21). Our laboratory has made progress in the disease and immune research of this clam (22–26). We found that MITF participates in the immune response to *Vibrio parahaemolyticus* infection and the expression of MITF was upregulated during *V. parahaemolyticus* infection in this clam (27). However, how MITF functions in the immune response in *M. petechialis* has not been investigated. Since the immune mechanism is quite different between vertebrates and invertebrates, we wondered if the immune signaling pathway regulated by MITF in clams is similar to that in vertebrates. In addition, to clarify the MITF's immune function in the clam immunity will contribute to our understanding in the clam immunity and benefit for the future clam resistance selection. In the present work, we identified *PO, CTSK*, and *BCL-2* as the downstream target genes of MITF. We analyzed the function of these genes, demonstrating that MpPO and MpCTSK can suppress the growth of *V. parahaemolyticus* and that MpBCL-2 is associated with the antiapoptotic activity of the haemocyte response to *V. parahaemolyticus* infection. This study revealed MpMITF as a transcriptional regulator of the innate immune system in the clam *M. petechialis*.

## Materials and Methods

### Experimental Clams, Bacterial Challenge, and Tissue Collection

Adult clams (43.7 ± 0.35 mm in shell length) were sampled from our laboratory-bred strains, which were cultured in a wild pond in Zhejiang province, China. Samples were acclimated in the laboratory for 1 week and supplied with filtered aerated seawater and microalgae. For bacterial challenge assays, approximately 100 clams were placed in a 200 L water tank to be challenged by immersion in a concentration of 1 × 10^7^ CFU mL^−1^ of *V. parahaemolyticus* (25). The challenge assay lasted for a sampling period of 13 days. Clams were randomly sampled and the hepatopancreases were dissected on days 0, 2, 4, 6, 8, 10, 11, and 13 after challenge, and then the hepatopancreases were preserved in liquid nitrogen before RNA extraction. There were five replicates for each time point.

### Total RNA Extraction and cDNA Synthesis

Total RNA was isolated using a total RNA Kit (Omega, USA) according to the manufacturer's instructions. The extracted RNA was quantified using a NanoDrop ND1000 spectrophotometer (Thermo Scientific, USA). The first-strand cDNA was synthesized from total RNA with a PrimeScript™ 1st Strand cDNA Synthesis Kit (TaKaRa, Japan) following the manufacturer's instructions. The cDNA used for quantitative real-time PCR was synthesized from total RNA using the PrimeScript™ RT reagent Kit with a gDNA Eraser (TaKaRa, Japan).

### RNA Interference (RNAi) of *MpMITF* in Clams

The cDNA fragment of *MpMITF* (GenBank: KY446553) was amplified using the T7 promoter-linked primers, MITF-T7-F and MITF-T7-R (Table 1), and was used as a template for the synthesis of the *MpMITF* double-stranded RNA (dsMITF). A fragment of the pEGFP vector plasmid (Clontech, USA) was amplified with EGFP-T7-F and EGFP-T7-R (Table 1) and used as a template for the synthesis of the *EGFP* double-stranded RNA (dsEGFP). The dsMITF and dsEGFP were synthesized and purified using a TranscriptAid T7 High Yield Transcription Kit (Thermo Scientific, USA). The quantity and integrity of the dsRNAs were determined using a NanoDrop ND1000 spectrophotometer (Thermo Scientific, USA) and agarose gel electrophoresis, respectively.

**Table 1:** Sequences of the primers used in this study.

**Primer**	**Sequence(5^**′**^-3^**′**^)**
MpCTSK-GSP1	GGACAGAGCCAAAGAACAACAG
MpCTSK-GSP2	CTTTGATGTCCGCCGAGATTAC
MpPO-GSP1	CGGAACAACTTGCCGTGTCTGC
MpPO-GSP2	AAACGGCAGCCACGAGGATT
MpBCL2-GSP1	TCATTCCATAGGGACTCTTC
MpBCL2-GSP2	GTCGTCCTACTACAATCACTTT
MpCTSK-P-F	CATGTAGTAGTAGTGGAGGC
MpCTSK-P-R	TCTAAAGATTGATCGAATGAG
MpPO-P-F	ACACCAAAGTTGACGGAACAGC
MpPO-P-R	ATCACTTGCCCGTCACCTCTAT
MpBCL2-P-F	CAATGCCCAGCAAGGGAGAAAT
MpBCL2-P-R	TCCCTTCCCGTCTTTCTCATAG
MITF-T7-F	GCGTAATACGACTCACTATAGGGCAAAGCCCAAAGAC GACGGA
MITF-T7-R	GCGTAATACGACTCACTATAGGGATGGACCACCAGAC GCTATCAC
EGFP-T7-F	GCGTAATACGACTCACTATAGGGAGCCATACCACATT TGTAGAGG
EGFP-T7-R	GCGTAATACGACTCACTATAGGGCGCTTTCTTCCCTT CCTTT
Actin-F	TTGTCTGGTGGTTCAACTATG
Actin-R	GACTGATTTCTTACGGATG
BCL-RT-F	GCACAGAACGGTTGAGAA
BCL-RT-R	TACGGAAACAAGACAAAGCT
CTSK-RT-F	ACATTTCCTCAATCCCTCAG
CTSK-RT-R	TTGGCTCTGTCCGTCTGT
PO-RT-F	TGGGACTCTACCCTAGATGACC
PO-RT-R	CGTGCCACCTGTACCAGAATAT
MpCTSK-F	GGAAAATATTTTCTTCCGTTGC
MpCTSK-R	GACAATCGGATAACTTGCCCAT
MpPO-F	ATGAAAATCCTCGTGGCTGC
MpPO-R	CTACCACGGCCAAAACGACT

Thirty clams used for RNAi experiments were randomly divided into two groups including the dsMITF-injected group (15 clams) and the dsEGFP-injected group (15 clams; negative control). The dsMITF/dsEGFP was injected into the hepatopancreas of each clam with a microsyringe. Each clam in the injected groups was injected with 10 μL of dsMITF or dsEGFP (3 μg/μL). The hepatopancreases of five clams in each group were collected at 12 and 48 h post-injection (hpi) and stored in liquid nitrogen before RNA extraction. Quantitative real-time PCR was used to test the efficiency of *MpMITF* knockdown and the expression of putative MpMITF downstream genes.

### Quantitative Real-Time PCR

Quantitative real-time PCR (qRT-PCR) was carried out using appropriate primers with an ABI 7500 fast Real-Time Thermal Cycler machine (Applied Biosystems, USA). β*-actin* was used as the internal reference to normalize the expression levels between samples (23). Primers used in this experiment are listed in Table 1. Three repeats of each sample were run in a 10 μl reaction volume containing 20 ng of template, 0.3 μM of each primer and 5 μl of QuantiNova SYBR Green PCR Master Mix. The PCR parameters were 95°C for 2 min, followed by 40 cycles of 95°C for 5 s and 60°C for 20 s. The 2^−ΔΔ*CT*^ method was used to analyse the relative gene expression levels (28).

### Cloning of *MpCTSK, MpPO*, and *MpBCL-2* Promoter Sequences

The *MpCTSK, MpPO* and *MpBCL-2* promoters were cloned using the Universal Genome Walker 2.0 Kit (Clontech, USA). First, GenomeWalker libraries were constructed using clam genomic DNA. The gene-specific primers, MpCTSK/MpPO/MpBCL-GSP1 and MpCTSK/MpPO/MpBCL-GSP2 (Table 1), were designed to produce single DNA fragments by nested PCR. The primary PCR parameters were set as follows: 7 cycles of 94°C for 25 s and 72°C for 3 min, then 32 cycles of 94°C for 25 s and 67°C for 3 min, followed by an additional 7 min at 67°C. The secondary PCR parameters were as follows: 5 cycles of 94°C for 25 s and 72°C for 3 min, followed with 20 cycles of 94°C for 25 s and 67°C for 3 min, with a final 7 min extension at 67°C. Finally, the whole DNA fragment was confirmed by PCR using the gene-specific primers, MpCTSK-P-F/R, MpPO-P-F/R, and MpBCL-P-F/R (Table 1), and sequencing. The E-boxes in the promoters of *MpCTSK, MpPO*, and *MpBCL-2* were predicted with the online software Promoter 2.0 (http://www.cbs.dtu.dk/services/Promoter/).

### Electrophoretic Mobility Shift Assays (EMSAs)

Full-length MpMITF protein was generated by *in vitro* transcription using the One-Step Human IVT kit (Thermo Scientific, USA) following the manufacturer's protocol. The MpMITF protein was confirmed by Western blot using an anti-MITF antibody (ABClonal, USA). EMSAs were carried out using the Lightshift® Chemiluminescent EMSA Kit (Thermo Scientific, USA). Briefly, binding reactions were conducted in binding buffer for 20 min at room temperature using MpMITF protein and biotin-labeled annealed double-stranded probes containing the E-box elements from the MpCTSK/MpPO/MpBCL-2 promoters. For the competition assays, unlabelled probes were added to the reaction mixture 15 min before adding the labeled probes. Then, the mixtures were loaded onto a 6% native polyacrylamide gel and electrophoresed at 4°C for 120 min at 90 V in 0.5 × TBE. Following electrophoresis, the gel was transferred to a positively charged nylon membrane (Solarbio, China) and the shifted bands were visualized by chemiluminescence according to the manufacturer's manual.

### Yeast One-Hybrid Assays

A yeast one-hybrid (Y1H) assay was performed using the Matchmaker^TM^ Gold Yeast One-Hybrid System (Clontech, USA) as described in the manufacturer's protocol. Briefly, the promoter sequences of *MpPO*/*MpCTSK*/*MpBCL-2* were cloned into the pAbAi vector, which contains an AbA resistance (*Ab*A^*r*^) reporter gene, to construct CTSK/PO/BCL-2*-*pAbAi. Then, the CTSK/PO/BCL-2*-*pAbAi plasmid was linearized using the BstBI restriction enzyme (NEB, USA) and integrated into the genome of the Y1HGold yeast strain. The minimal inhibitory concentration of Aureobasidin A (AbA) for the yeast strain was confirmed using 300 ng/ml. Additionally, the full coding sequence of *MpMITF* was cloned into the pGADT7 vector to construct MITF-pGADT7, which expressed MITF fusion proteins containing the yeast GAL4 transcriptional activation domain (AD). The MITF-pGADT7 vector was transformed into the yeast strain containing CTSK/PO/BCL-2*-*pAbAi and screened on SD/-Leu/AbA plates. When the MITF protein binds to the *MpPO*/*MpCTSK*/*MpBCL-2* promoter sequences, the GAL4 AD will activate expression of *Ab*A^*r*^, allowing the cells to grow on media containing the AbA antibiotic.

### Expression, Purification, and Identification of MpCTSK and MpPO

To express the recombinant MpCTSK and MpPO proteins, the coding sequences of *MpCTSK* (GenBank: MK294530) and *MpPO* were separately amplified by the primer pairs MpCTSK-F/R and MpPO-F/R (Table 1) and cloned into a pET30a or pGEX-4T-1 expression vector, respectively. The generated expression plasmids were transformed into BL21 (DE3) pLysS Chemically Competent Cells (TransGen Biotech, China) for overexpression. After induction with a final concentration of 0.2 mM isopropyl-b-d-thio-galactoside (IPTG) at 28°C for 6 h, the bacteria were pelleted by centrifugation at 12,000 rpm for 10 min. The whole cell pellet suspensions were disrupted in PBS by sonication in an ice bath and then centrifuged at 12,000 rpm for 10 min. The recombinant MpCTSK and MpPO fusion proteins were purified with the His-Trap and GST-Trap affinity columns (GE Healthcare, Germany), respectively.

The purified recombinant MpCTSK and MpPO proteins were analyzed via 12% SDS-PAGE (GenScript, China). After electrophoresis, the gel was stained with Coomassie brilliant blue R250. In addition, the purified MpCTSK and MpPO proteins were identified by Western blot. Briefly, the purified recombinant protein samples were separated by SDS-PAGE (12%) and transferred onto a PVDF membrane. The membrane with transferred proteins was immersed in 5% non-fat milk in TBST for 2 h at room temperature and subsequently incubated with primary rabbit anti-cathepsin K or rabbit anti-TYR antibodies (ABClonal, USA; 1:1,000) at 4°C overnight. After washing three times for 10 min with TBST, the membranes were incubated with a diluted secondary goat anti-rabbit IgG-HRP antibody (ZSGB-BIO, China; 1:5,000). The target protein signals were detected with an Enhanced HRP-DAB Chromogenic Substrate Kit (Tiangen, China).

### Antimicrobial Activity Assays

Antimicrobial activity assays were performed to detect the antimicrobial activities of the purified recombinant MpCTSK and MpPO proteins *in vitro*. The purified recombinant MpCTSK (r MpCTSK) and MpPO (rMpPO) proteins were obtained in section Expression, Purification, and Identification of MpCTSK and MpPO. The antimicrobial activity of purified rMpCTSK and rMpPO was tested in 96-well microtiter plates. In detail, rMpCTSK and rMpPO were diluted with PBS to 1 μM. PBS was used as the blank control. *Vibrio parahaemolyticus* as well as *Staphylococcus aureus* and *Micrococcus luteus* were cultured and collected by centrifugation at 800 *g* for 10 min. After washing twice with PBS, the bacteria were resuspended at a concentration of 1 × 10^5^ CFU mL^−1^ in PBS. A volume of 100 μL of bacterial suspensions was mixed with 100 μL of 1 μM rMpCTSK, rMpPO or an equal volume of PBS. The 96-well microtiter plate was incubated at 30°C for 24 h and the absorbance of the plate was measured every 8 h at an OD of 600 nm. The experiment was repeated three times.

The bacterial feedback inhibition loop was examined to verify the inhibitory activity. In brief, aliquots of 50 μL bacterial suspensions of *V. parahaemolyticus* and *S. aureus* diluted to 1 × 10^5^ CFU mL^−1^ were spread on an agar plate. On each plate of *V. parahaemolyticus* or *S. aureus*, 10 μL of 1 μM rMpCTSK and rMpPO were spotted separately using filter papers. An equal volume of kanamycin was spotted as a positive control, while a GST-tagged protein/His-tagged protein and PBS were spotted as negative controls. Bacteria were grown on the agar plate overnight at 30 °C. Each experiment was performed with three repeats.

### Apoptosis Assay

An apoptosis assay was performed to gain insights into the role of MpBCL-2 on the clam haemocytes apoptosis. Haemocytes extracted from 20 healthy clams were mixed and divided equally into two groups, i.e., the ABT199-treated group and control group. Each group was sub-divided into three replicates. The haemocytes in the ABT199-treated group were cultured in L15 medium (Solarbio, China) added with a BCL-2 specific inhibitor, ABT199 (Beyotime biotechnology, China) at 18 °C for 12 h, while the heamocytes in the control group were cultured in L15 medium added with the equal volume of DMSO at 18°C for 12 h. Then, these haemocytes were applied to an apoptosis assay. In brief, the haemocytes were washed three times with L15 medium and then suspended in L15 medium to a final concentration of 1 × 10^6^ cells mL^−1^. The suspended haemocytes were subjected to flow cytometry (BD Biosciences, USA) for apoptosis rate detection using the Alexa Fluor® 488 annexin V/Dead Cell Apoptosis kit (Invitrogen, USA).

In addition, the influence of *V. parahaemolyticus* challenge on the clam haemocytes apoptosis was also detected. Briefly, 50 μL of *V. parahaemolyticus* with a concentration of 5 × 10^6^ CFU mL^−1^ was injected into the adductor of thirty clams individually. These *Vibrio*-injected clams were cultivated in a 50 L water tank for 48 h, during which the haemocytes were extracted using syringes from adductors of six clams at 0, 12, 24, and 48 hpi for apoptosis rate analysis by flow cytometry using the Alexa Fluor® 488 annexin V/Dead Cell Apoptosis kit.

### Statistical Analysis

All data are presented as the means ± S.D. Statistically significant differences were analyzed by one-way ANOVA using SPSS 19.0 software. The significance level was set as *P* < 0.05.

## Results

### Knockdown of MpMITF Influences the Expression of Downstream Genes

**Figure 1:**
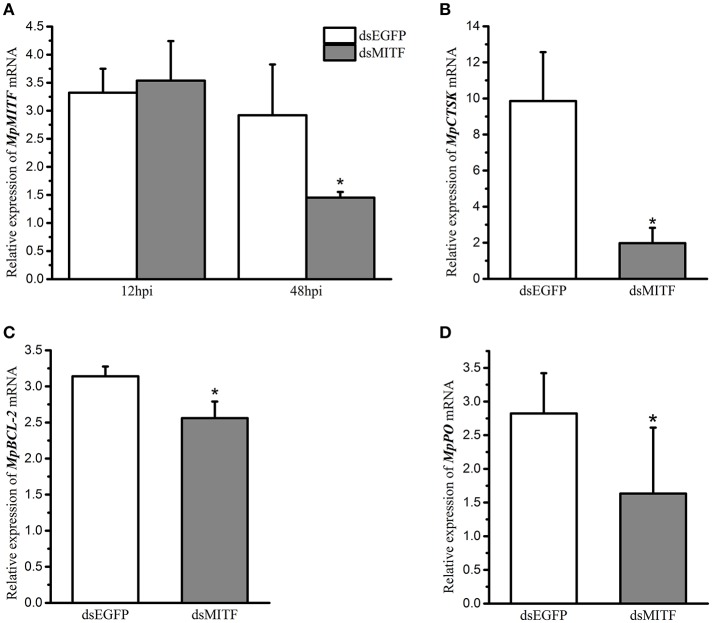
Relative mRNA expression of genes in RNAi experiments by qRT-PCR. **(A)** Relative mRNA expression of *MpMITF* in clams injected with dsMITF/dsEGFP at 12 and 48 hpi. **(B–D)** Relative mRNA expression of potential MITF targets in clams injected with dsMITF/dsEGFP at 48 hpi. Error bars represent the SD. Asterisks (*) represent a significant difference between the dsMITF-injected group and the dsEGFP-injected group (*P* < 0.05).

An RNAi experiment was used to identify the potential downstream genes of MpMITF. The efficiency of interference was determined by qRT-PCR. The qRT-PCR results showed that, compared to the dsEGFP-injected group, the mRNA expression of *MpMITF* in the dsMITF-injected group was significantly downregulated at 48 hpi (*P* < 0.05) (Figure 1A), indicating that the RNAi worked. The change in mRNA expression for a series of putative MpMITF downstream genes, including *MpCTSK, MpPO, MpBCL-2, MpCDK2, MpPerforin, MpNDST*, and *MpTryptase*, was measured in the *MpMITF*-suppressed individuals by qRT-PCR. The results showed that, compared to the dsEGFP-injected group, the expression of *MpCTSK, MpPO*, and *MpBCL-2* was significantly reduced at 48 hpi in the dsMITF-injected group (*P* < 0.05) (Figures 1B–D). No significant differences were observed in the expression of *MpCDK2, MpPerforin, MpNDST*, and *MpTryptase* before and after RNAi (Supplementary Figures 1A–D). These results suggested that *MpCTSK, MpPO*, and *MpBCL-2* are the potential downstream targets of MpMITF.

### MpMITF Binds to the Promoters of *MpCTSK*/*MpPO*/*MpBCL-2*

**Figure 2:**
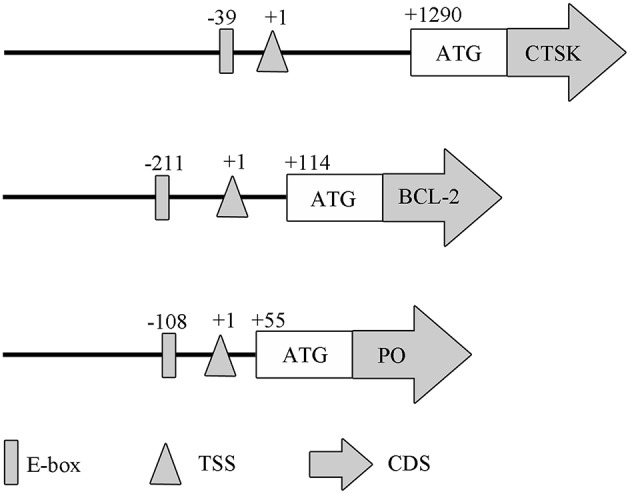
Domain architectures of the *MpPO, MpCTSK*, and *MpBCL-2* promoters, predicted using their promoter sequences. The positions of the E-box, transcription start site (TSS), initiation codon (ATG), and coding sequence (CDS) are annotated.

**Figure 3:**
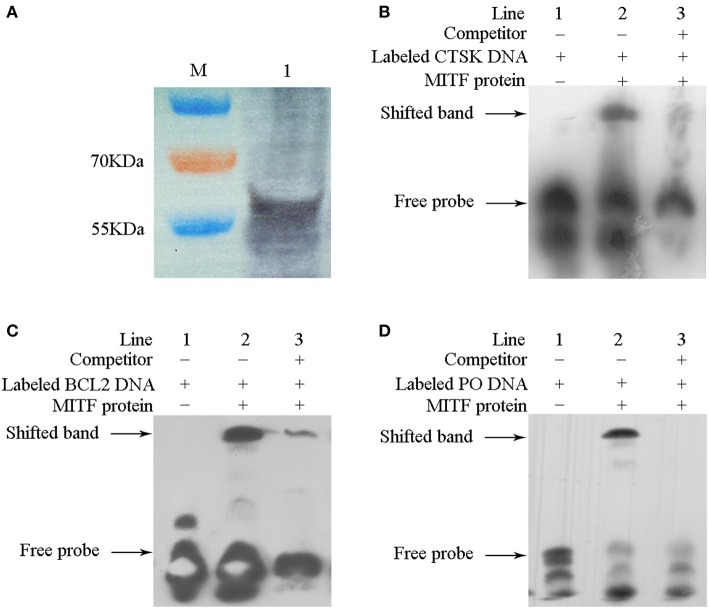
Interaction between MpMITF protein and the *MpPO/MpCTSK/MpBCL-2* promoters. **(A)** The MpMITF protein synthesized by *in vitro* transcription and shown by Western blot. **(B–D)** Incubation of biotin-labeled *MpPO/MpCTSK/MpBCL-2* promoter probes with the MITF protein formed a strong shifted band (line 2) compared to labeled *MpPO/MpCTSK/MpBCL-2* promoter probes alone (line 1). Binding was prevented by the addition of excess unlabelled competitor DNA (line 3). The shifted band and free probe are marked with the arrowheads.

To evaluate if MpMITF directly binds to the *MpCTSK/MpPO/MpBCL-2* promoters, we performed EMSAs using MpMITF recombinant protein and DNA fragments of the *MpCTSK/MpPO/MpBCL-2* promoters. The *MpCTSK, MpPO*, and *MpBCL-2* promoters were cloned and all three promoters were found to contain the E-box (CANNTG) sequence (Figure 2). The MpMITF protein with a molecular weight of 57 kDa was verified by Western blot (Figure 3A). Signal detection in EMSAs is based on biotin-labeled DNA in complex with proteins moving more slowly during electrophoresis forming a shifted band. Compared to the control reactions (Figures 3B–D, line 1), a shifted band was observed when labeled *MpCTSK/MpPO/MpBCL-2* DNA and MITF protein were added together, indicating that the *MpCTSK/MpPO/MpBCL-2* promoters formed a covalent DNA-protein complex with the MpMITF protein (Figures 3B–D, line 2). However, the band shift was prevented by the addition of excess unlabelled DNA (competitor DNA) (Figures 3B–D, line 3). The EMSA results showed that MpMITF specifically binds to the promoter regions of *MpCTSK, MpPO*, and *MpBCL-2*, further suggesting that *MpCTSK, MpPO*, and *MpBCL-2* are transcriptionally regulated by MpMITF.

### MpMITF Activates the Expression of *MpCTSK*/*MpPO*/*MpBCL-2*

**Figure 4:**
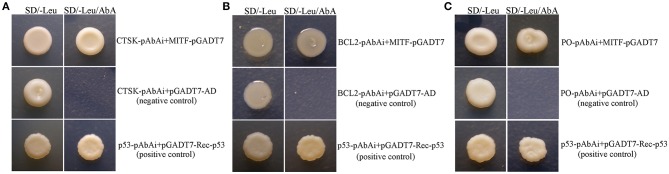
**(A–C)** Yeast one-hybrid analysis of MpMITF binding to the *MpPO/MpCTSK/MpBCL-2* promoters. The MpPO/MpCTSK/MpBCL-2-pAbAi plasmid together with the pGADT7-MITF effector plasmid were cotransformed into Y1HGold cells to validate the interaction. The positive control was generated by cotransforming the pGADT7-Rec-p53 and p53-pAbAi plasmids into Y1HGold cells. The Y1HGold strain cotransformed with the MpPO/MpCTSK/MpBCL-2-pAbAi and pGADT7-AD plasmids was used as a negative control. The normal growth of all Y1HGold yeast strains on SD plates without Leu (SD/-Leu) indicated that the yeast growth status was healthy (left). Only the strains containing the effector plasmid pGADT7-MITF and the positive control showed growth on SD/-Leu containing 300 ng/ml aureobasidin A (SD/-Leu/AbA, right), suggesting a specific interaction between the *MpPO/MpCTSK/MpBCL-2* promoters and MpMITF.

To test if MpMITF could regulate the expression of *MpCTSK, MpPO*, and *MpBCL-2*, yeast one-hybrid assays were performed. In this analysis, the AbA resistance (*Ab*A^*r*^) reporter gene was not activated when the Y1HGold yeast strain was cotransformed with the CTSK/PO/BCL-2*-*pAbAi and empty pGADT7-AD plasmids because the transcription of CTSK/PO/BCL-2 was not activated, resulting in no growth on the SD/-Leu medium with AbA (Figure 4, negative control). By contrast, the Y1HGold yeast strain cotransformed with the MITF-pGADT7 and CTSK/PO/BCL-2*-*pAbAi plasmids could grow on the SD/-Leu medium with AbA (Figure 4), indicating that MpMITF activated the expression of CTSK/PO/BCL-2 and *Ab*A^*r*^, which allowed the cells to grow on media containing the AbA antibiotic. These results suggested that MpMITF can directly activate the expression of *MpCTSK, MpPO*, and *MpBCL-2*.

### *MpCTSK, MpPO* and *MpBCL-2* Respond to *V. parahaemolyticu*s Infection

**Figure 5:**
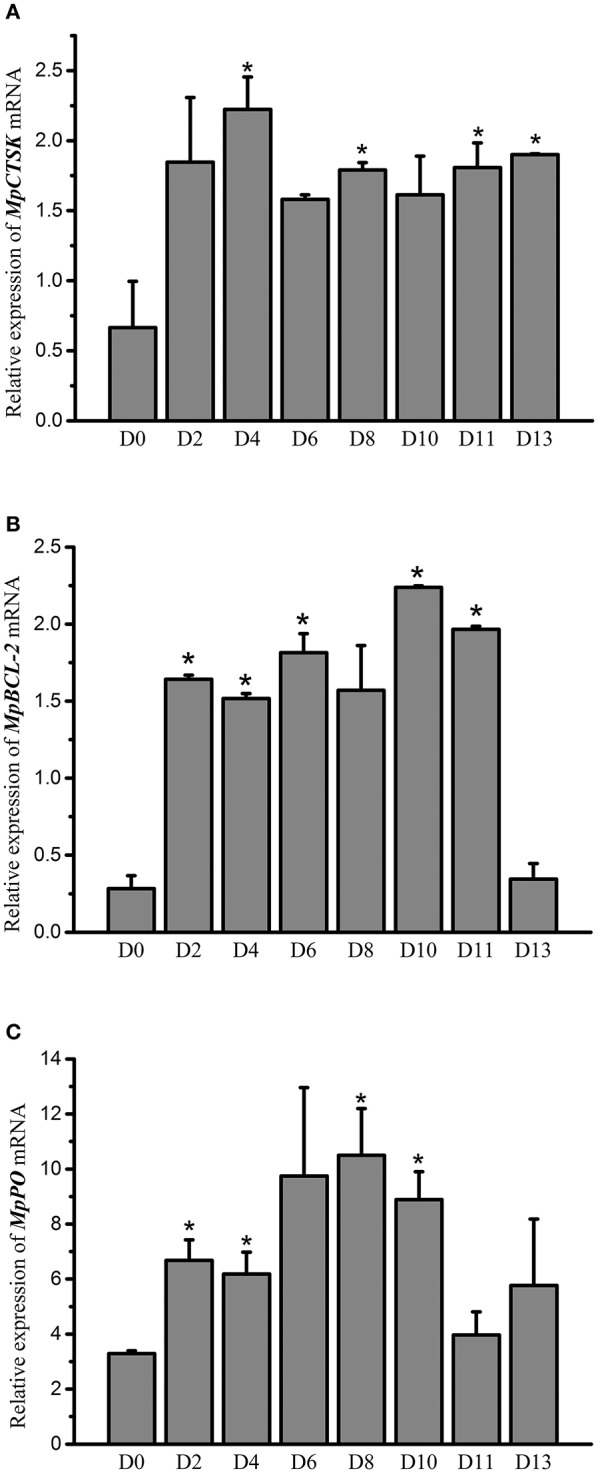
Relative mRNA expression of MpCTSK **(A)**/MpBCL-2 **(B)**/MpPO **(C)** in the hepatopancreases of *M. petechialis* at 0–13 dpi after immersion in *V. parahaemolyticus* by qRT-PCR. Error bars represent the SD. The asterisk (*) represents significant differences found when compared to 0 dpi (*P* < 0.05).

To investigate if the expression of the MpMITF downstream genes was influenced by *V. parahaemolyticus* challenge, the mRNA expression levels of *MpCTSK, MpPO*, and *MpBCL-2* were measured by qRT-PCR. The tissue distributions of *MpCTSK, MpPO* and *MpBCL-2* in the hepatopancreas, mantle, foot, gill, haemolymph and adductor muscle were analyzed. The results showed that all of these genes were widely expressed in the six tissues (Supplementary Figure 2). The hepatopancreas is the main immune organ in clams and thus was selected for the further study (29). A *V. parahaemolyticus* immersion challenge was performed to investigate the *MpCTSK, MpPO* and *MpBCL-2* expression response to *V. parahaemolyticus* infection in the clam. As Figure 5A shows, the mRNA expression level of *MpCTSK* was increased at 2 d post infection (dpi), reached the highest level at 4 dpi, and remained at a high level until 13 dpi. Significant differences were detected at 4, 8, 11, and 13 dpi, compared to 0 dpi (*P* < 0.05). The mRNA expression level of *MpBCL-2* was increased at 2 dpi, peaked at 10 dpi, and then decreased to base levels at 13 dpi. Significantly different mRNA expression levels were detected at 2, 4, 6, 10, and 11 dpi compared with those at 0 dpi (*P* < 0.05, Figure 5B). Similarly, the expression of *MpPO* was significantly increased at 2, 4, 8 and 10 dpi compared with 0 dpi (*P* < 0.05, Figure 5C). These results indicated that *MpCTSK, MpPO* and *MpBCL-2* are involved in the immune response toward *V. parahaemolyticus* infection.

### MpCTSK and MpPO Inhibit the Growth of Bacteria

**Figure 6:**
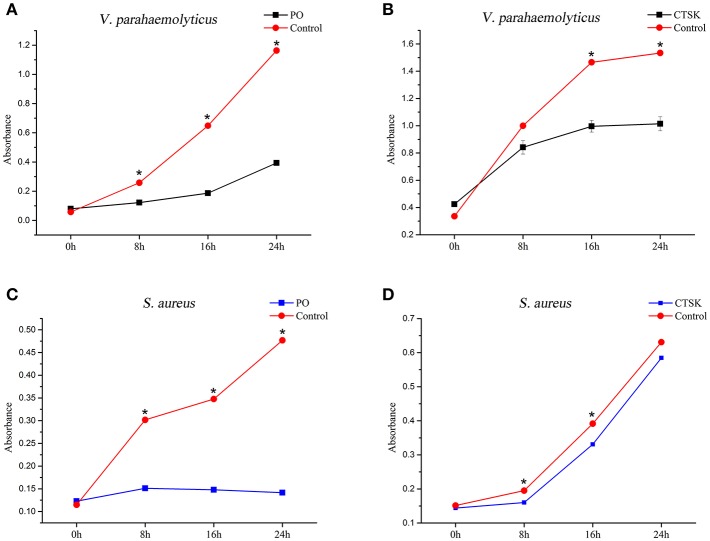
Antibacterial activities of rMpPO and rMpCTSK evaluated by the minimal inhibitory concentration method. **(A,B)** The inhibition of *V. parahaemolyticus* growth at 0, 8, 16, and 24 h post rMpPO- or rMpCTSK-addition. **(C,D)** The inhibition of *Staphylococcus aureus* growth at 0, 8, 16, and 24 h post rMpPO- or rMpCTSK-addition. Error bars represent the SD. Asterisks (*) represent a significant difference between groups given rMpPO/rMpCTSK and the control group (*P* < 0.05).

**Figure 7:**
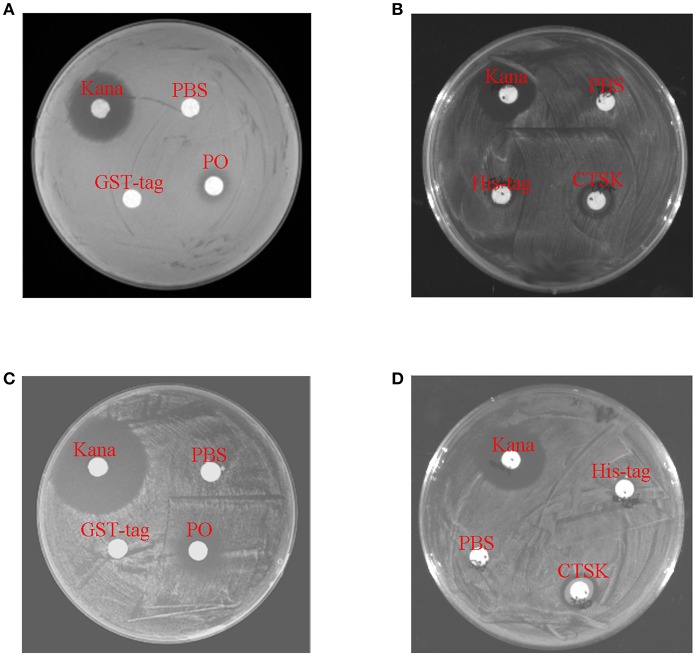
Antibacterial activities of rMpPO and rMpCTSK evaluated by the bacterial inhibition loop method. **(A,B)** The growth of *V. parahaemolyticus* on an LB agar plate with rMpPO or rMpCTSK added. **(C,D)** The growth of *Staphylococcus aureus* on an LB agar plate with rMpPO or rMpCTSK added. Kanamycin was used as positive control. GST-tagged protein/His-tagged protein and PBS were used as negative controls.

To determine the roles of the downstream genes MpCTSK and MpPO in response to bacteria, recombinant MpCTSK and MpPO proteins were produced in a prokaryotic expression system. SDS-PAGE results demonstrated that the molecular weights of the recombinant MpCTSK (rMpCTSK) and recombinant MpPO (rMpPO) were approximately 39 and 107 kDa, respectively (Supplementary Figures 3A,B), consistent with the mature proteins predicted from the cDNA sequences. The protein bands were confirmed by Western blot with specific anti-cathepsin K or anti-PO antibodies (ABClonal, USA) (Supplementary Figures 3C,D). The antibacterial activities of rMpCTSK and rMpPO were then evaluated by the minimal inhibitory concentration method. As shown in Figures 6A,B, the growth of *V. parahaemolyticus* was significantly suppressed at 8 h, 16 h and 24 h when rMpCTSK or rMpPO were added, compared to PBS (*P* < 0.05). Furthermore, the growth of another pathogen, *Staphylococcus aureus*, was also inhibited when rMpCTSK or rMpPO were added, while the growth of *Micrococcus luteus* was not (Figures 6C,D). In addition, the antibacterial properties of rMpCTSK and rMpPO were also detected by an agar diffusion test. The results showed that rMpCTSK and rMpPO displayed strong antimicrobial activities against *V. parahaemolyticus* and *S. aureus*, while no antibacterial properties were detected using filter paper with PBS or GST/His-tagged protein (Figures 7A–D). All of these results demonstrated that MpCTSK and MpPO have strong antibacterial activities against *V. parahaemolyticus* and *S. aureus*.

### MpBCL-2 Inhibits the Apoptotic Activity of Haemocytes During Immune Defense

**Figure 8:**
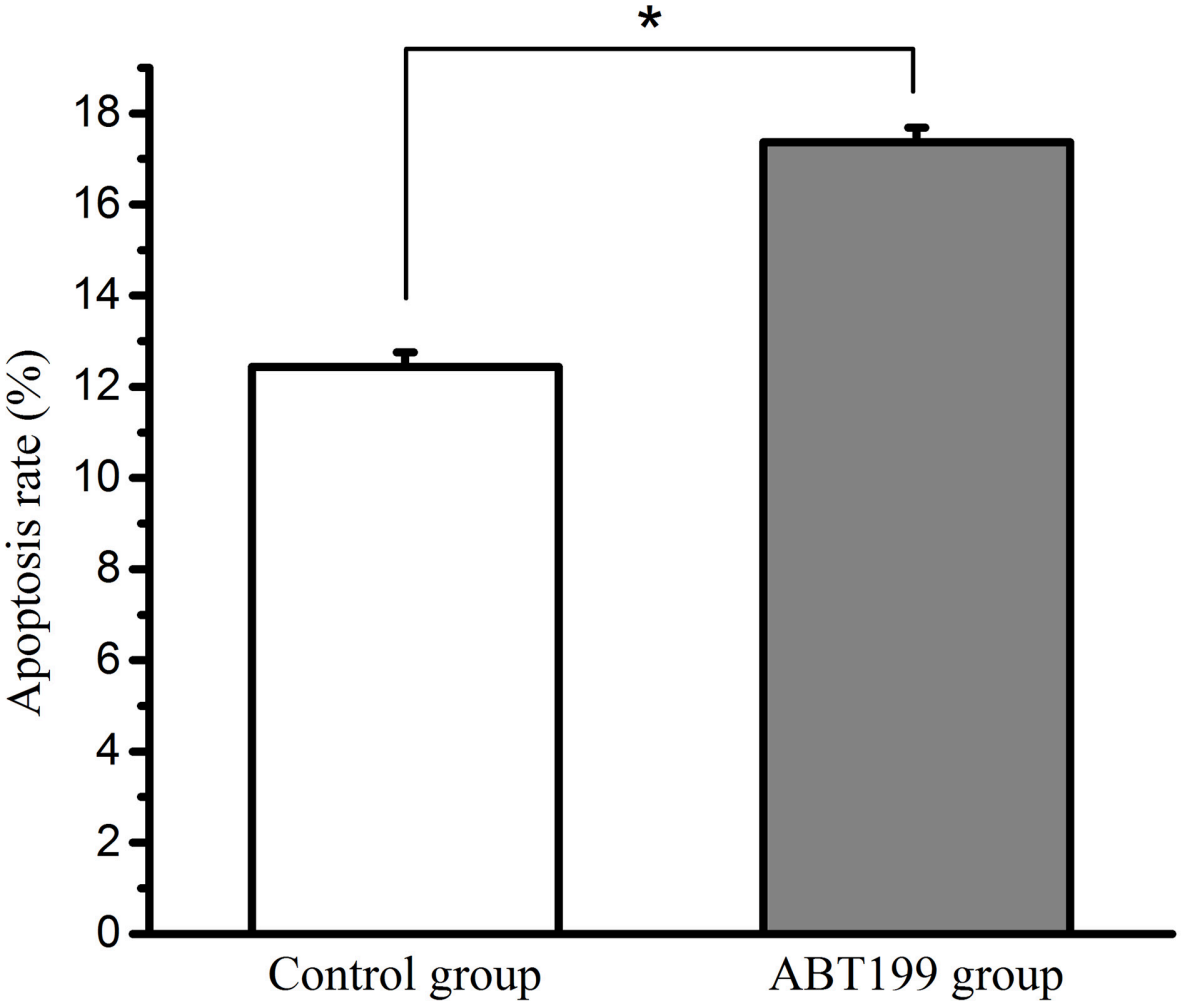
The apoptosis rate of haemocytes in the ABT199-treated group and the control group. Error bars represent the SD. Asterisks (*) represent a significant difference between the ABT199-treated group and the control group (*P* < 0.05).

Our results showed that the haemocyte apoptosis rate was increased post-*V. parahaemolyticus* injection (Supplementary Figure 4), indicating that *Vibrio*-induced stress promoted the apoptosis rate in clams. The relationship between BCL-2 and the apoptosis rate was then evaluated. To inhibit the activity of BCL-2, the specific inhibitor, ABT199, was used in this study (30). The haemocytes were treated with the BCL-2 inhibitor for 12 h (ABT199, diluted with DMSO), while the haemocytes of the control group were treated with equal volume of DMSO. The data (Figure 8) showed that the percent of apoptotic cells in the ABT199-treated group (17.4%) was significantly higher than that in the control group (12.4%) (*P* < 0.05), implying that BCL-2 inhibits the apoptosis of haemocytes. Our results have shown that the expression of MpBCL-2 is significantly increased after *V. parahaemolyticus* challenge and that *V. parahaemolyticus* stress promotes the apoptosis rate in clams. These findings suggest that BCL-2 is involved in the immune response to *V. parahaemolyticus* challenge through the inhibition of haemocyte apoptosis in clams.

## Discussion

The microphthalmia-associated transcription factor is a crucially important transcription factor that regulates a series of downstream genes. In our previous study, we have found that *MpMITF* was associated with the immune defense of the clam *M. petechialis* (27). In this research, we investigated how MpMITF plays a key role in immune function, acting as a transcription factor in clams. According to the existing reports on MITF, we selected several MITF-regulated immune-related downstream genes, including *PO, CTSK, CDK2, NDST, tryptase, perforin* and *BCL-2*, and determined whether these genes were also regulated by MpMITF in clams. In addition, we analyzed the function of these downstream genes in the immune response of molluscs, revealing the regulatory mechanisms of MpMITF in the innate immunity of clams.

The downstream genes transcriptionally regulated by MITF have been widely reported in vertebrates but rarely reported in invertebrates. In this study, we first knocked down the expression of MpMITF and evaluated the expression levels of putative downstream genes including *PO, CTSK, CDK2, NDST, tryptase, perforin* and *BCL-2*, according to previous studies (9–15). Our results showed that among these seven genes, the expression of *MpPO, MpCTSK*, and *MpBCL-2* was significantly reduced after knockdown of MpMITF (Figure 1). A previous study indicated that mutated MITF represses the transcription of the tyrosinase gene (31). Overexpression of MITF dramatically upregulated the expression of CTSK in human osteoclasts (10). Moreover, MITF was reported to upregulate the expression of BCL-2 in tumor cells (15). Our results indeed confirmed that several genes (*PO, CTSK*, and *BCL-2*) positively regulated by MITF in other species were also strongly upregulated by MpMITF, suggesting that they were downstream genes of MpMITF in clams.

For the potential downstream genes of MpMITF selected by RNAi, EMSAs were used to investigate their direct interactions with MpMITF. Downstream genes transcriptionally regulated by MITF are mainly governed by the bHLH-LZ domain of MITF (32). We have previously identified a bHLH-LZ structure in the MpMITF protein sequence (27). In this study, we cloned the promoter sequences of *MpPO, MpBCL-2*, and *MpCTSK* and were able to detect an E-box sequence in all three of these genes (Figure 2). The E-box sequence is an essential element for the MITF protein to bind to Hughes et al. (5). Our EMSA results showed that a shifted band was detected when labeled *MpCTSK/MpPO/MpBCL-2* DNA and MITF protein were added together. Additionally, this shifted band was prevented by the addition of excess unlabelled DNA (Figure 3). These results indicated that MpMITF directly binds to the promoter regions of *MpCTSK, MpPO*, and *MpBCL-2*. This binding preference is consistent with published data demonstrating that MITF binds to E-box motifs. For example, Bently et al. showed that MITF transactivated the *PO* promoter via the E-box (9). *BCL-2* and *CTSK* are also direct MITF target genes via an E-box motif CATGTG in their promoters (33, 34).

Based on our results, MpMITF appeared to modulate downstream gene expression by binding to their promoters; our Y1H results further confirmed that the transcription of potential downstream genes was directly regulated by MpMITF. Our data showed that cotransformation of MpPO/MpCTSK/MpBCL-2-pAbAi and MITF-pGADT7 could activate the expression of the AbA resistance (*Ab*A^*r*^) reporter gene, leading to the growth of the yeast strain on SD/-Leu medium with AbA. However, the Y1HGold yeast strain cotransformed with MpPO/MpCTSK/MpBCL-2-pAbAi and empty pGADT7 could not grow on SD/-Leu medium with AbA because the CTSK/PO/BCL-2 promoters were not bound and thus the expression of *Ab*A^*r*^ could not be activated, further verifying that MpMITF could directly activate the expression of *MpCTSK, MpPO* and *MpBCL-2* (Figure 4). Similarly, other studies have also indicated that overexpression of MITF could activate the expression of CTSK and PO (35, 36). All of these results support the conclusion that MpMITF can directly regulate the transcription of *MpCTSK, MpPO*, and *MpBCL-2*.

*MpPO, MpCTSK*, and *MpBCL-2* have been suggested to be the downstream genes of MpMITF based on the results of RNAi, EMSA and Y1H assays, but whether *MpPO, MpCTSK*, and *MpBCL-2* are involved in the immune defense of clams has not been investigated. In the present study, we showed that during *V. parahaemolyticus* challenge, the mRNA expression of *MpCTSK* was significantly upregulated and remained at a high level until 13 dpi, indicating its participation in the immune defense against microbial infection (Figure 5A). This notion is supported by the report that CTSK expression was significantly increased post-injection with LPS in the olive flounder *Paralichthys olivaceus* (37). Meanwhile, the mRNA expression of *MpPO* and *MpBCL-2* was significantly increased at 2 dpi and then decreased back to base level at 13 dpi, however, their initially upregulated expression indicated their response to *V. parahaemolyticus* infection as well (Figures 5B,C). Consistently, PO activity has been found to be significantly increased after LPS stimulation, as described by Zhou et al. (38). Additionally, increased levels of BCL-2 gene expression have also been observed after *V. parahaemolyticus* challenge in the orange-spotted grouper *Epinephelus coioides* (39).

Since *MpCTSK, MpPO*, and *MpBCL-2* were associated with the immune defense against *V. parahaemolyticus* infection, it was necessary to figure out the immune functions of *MpCTSK, MpPO*, and *MpBCL-2* in the immune response to *V. parahaemolyticus* challenge. Here, we observed that the purified recombinant MpPO protein inhibited the growth of *V. parahaemolyticus* and *S. aureus* (Figures 6, 7). This was consistent with the notion that PO could inhibit the growth of *Vibrio* in the scallop *Chlamys farreri*, discussed previously by Xing et al. (40). Bacterial growth inhibition was also observed in the presence of PO in *Crassostrea gigas* (41). Additionally, PO has been characterized in the Manila clam *Venerupis philippinarum* and was involved in the immune response to bacterial infection (42–44). Moreover, the purified recombinant MpCTSK protein also inhibited the growth of *V. parahaemolyticus* and *S. aureus* (Figures 6, 7). Although CTSK has been reported to participate in innate immunity, its specific function has not been totally investigated to our knowledge (45). The present study is the first to explore the antibacterial value of CTSK in molluscs.

Finally, the antiapoptotic capability of MpBCL-2 was assessed in this study. We found that *V. parahaemolyticus* injection could promote the apoptosis of haemocytes (Supplementary Figure 4). Apoptosis is involved in many fundamental processes of the immune system, such as the regulation of the immune defense (46). The induction of apoptosis caused by pathogens has been described in other molluscs. For instance, haemocyte apoptosis in the Pacific oyster *Crassostrea gigas* was induced during infection with *Planococcus citreus* (47). To clarify the relationship between MpBCL-2 and cell apoptosis, the apoptosis rate of haemocytes was analyzed when MpBCL-2 was disrupted. Our findings showed a significant increase in the apoptosis rate of the BCL-2-interfered group compared to the control group, indicating an important role for MpBCL-2 in the immune system of clams (Figure 8). These results are in agreement with the previous report that the percentage of apoptotic cells declined when BCL-2 was overexpressed (48). Antonsson et al. found that BCL-2 could bind to proapoptotic proteins (e.g., Bax and Bim) to sequester these proapoptotic proteins in a neutralized state and prevent them from inducing apoptosis (49). The results presented here established the antiapoptotic role of MpBCL-2 and provided fundamental support to the suggested involvement of MpBCL-2 in the immune defense against *V. parahaemolyticus*.

In conclusion, we discovered that *MpPO, MpCTSK*, and *MpBCL-2* are downstream genes of MpMITF. MpMITF can directly bind to the promoters of *MpPO, MpCTSK*, and *MpBCL-2* and activate their transcriptional expression. MpPO and MpCTSK were found to suppress the growth of *V. parahaemolyticus* and MpBCL-2 was demonstrated to be associated with antiapoptotic activity in haemocytes. Our findings identify the clam immune-related genes regulated by MITF and reveal the immune regulatory mechanism of MITF in clams, which is unknown in invertebrates before. Some genes regulated by MITF in vertebrates were not regulated by MpMITF in clams, implying that the immune regulatory mechanism of MITF in clams differs from that in vertebrates. Overall, this study clarifies the role of MITF signaling pathway in the innate immunity of the clam *M. petechialis*.

## Ethics Statement

All experiments involving animals reported in this study were approved by the Ethics Committee of the Institute of Oceanology, Chinese Academy of Sciences.

## Author Contributions

BL, XY, HW, and SZ: conceived and designed the expriments. SZ, XY, and JY: performed the experiments. SZ and XY: analyzed the data. SZ, XY, and BL: wrote the paper.

### Conflict of Interest Statement

The authors declare that the research was conducted in the absence of any commercial or financial relationships that could be construed as a potential conflict of interest.
