# Supplementary material for: MITF Regulates Downstream Genes in Response to Vibrio parahaemolyticus Infection in the Clam Meretrix Petechialis
Source: Front Immunol. 2019 Jul 4;10:1547. doi: 10.3389/fimmu.2019.01547 (PMC6620822; doi:10.3389/fimmu.2019.01547)
Supplement: Supplementary file 1 [file Data_Sheet_1.doc]

Supplementary Material

## 1 Supplementary Figures


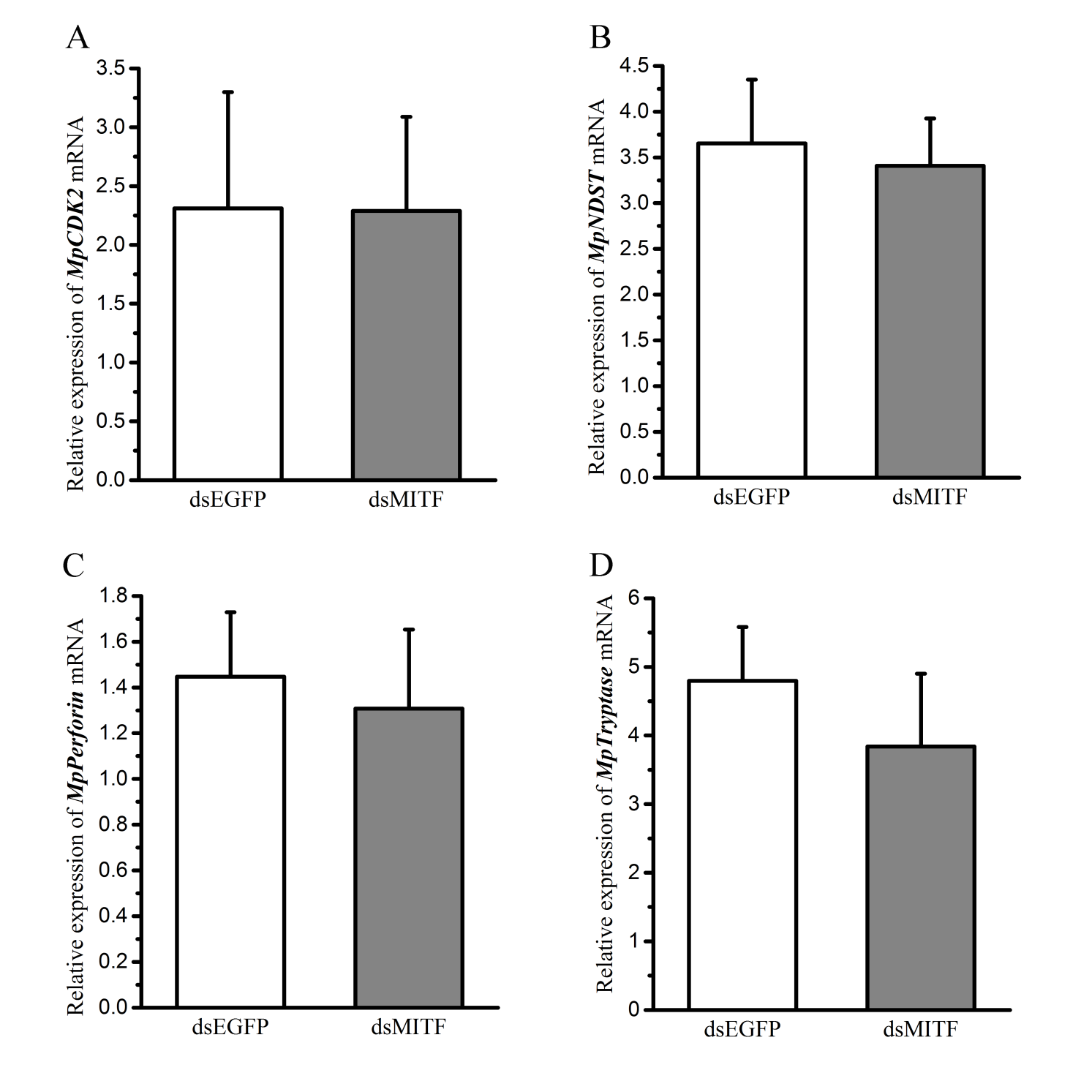


**Supplementary Figure 1.** Relative mRNA expression of *MpCDK2* **(A)/***MpNDST*

**(B)/***MpPerforin* **(C)**/*MpPerforin* **(D)** in clams injected with dsMITF/dsEGFP at 48 hpi..


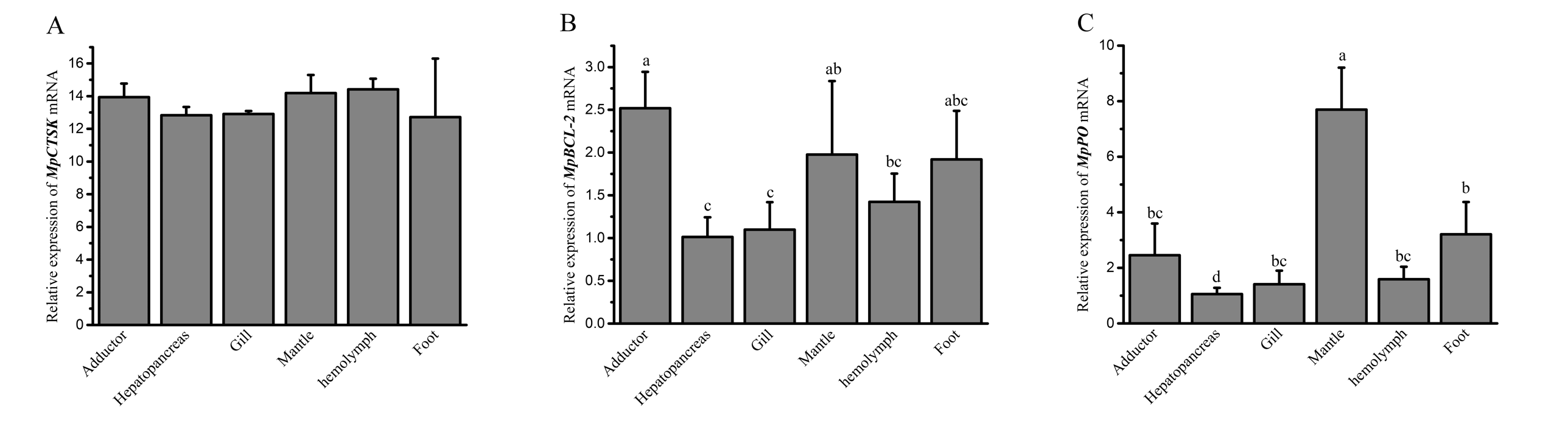


**Supplementary Figure 2.** Relative mRNA expression of *MpCTSK* **(A)***/MpBCL-2* **(B)***/MpPO* **(C)** in different tissues of *M. petechialis*. Error bars represent the SD. The different lowercase represents significant differences (*P* < 0.05).


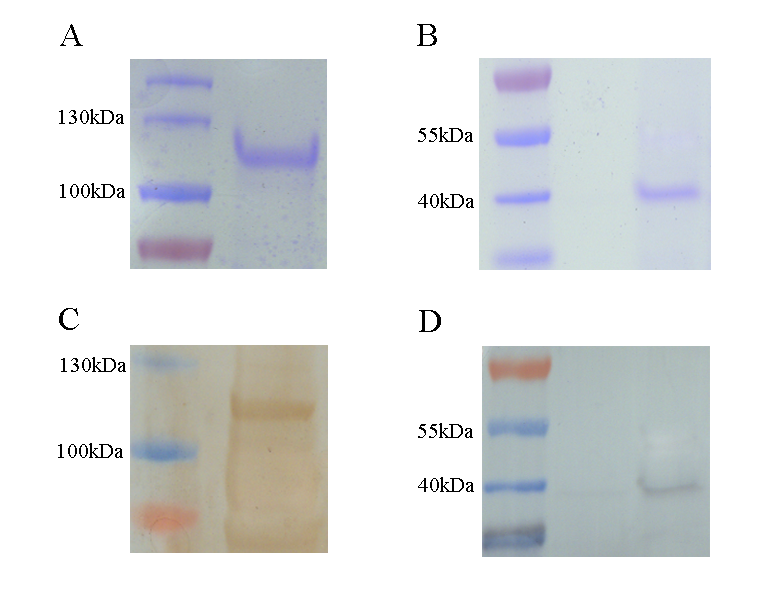


**Supplementary Figure 3.** The identification of the recombinant MpPO and MpCTSK proteins. **(A)** The recombinant MpPO protein stained with Coomassie brilliant blue R250. **(B)** The recombinant MpCTSK protein stained with Coomassie brilliant blue R250. **(C)** The recombinant MpPO protein shown by Western blot. **(D)** The recombinant MpCTSK protein shown by Western blot.


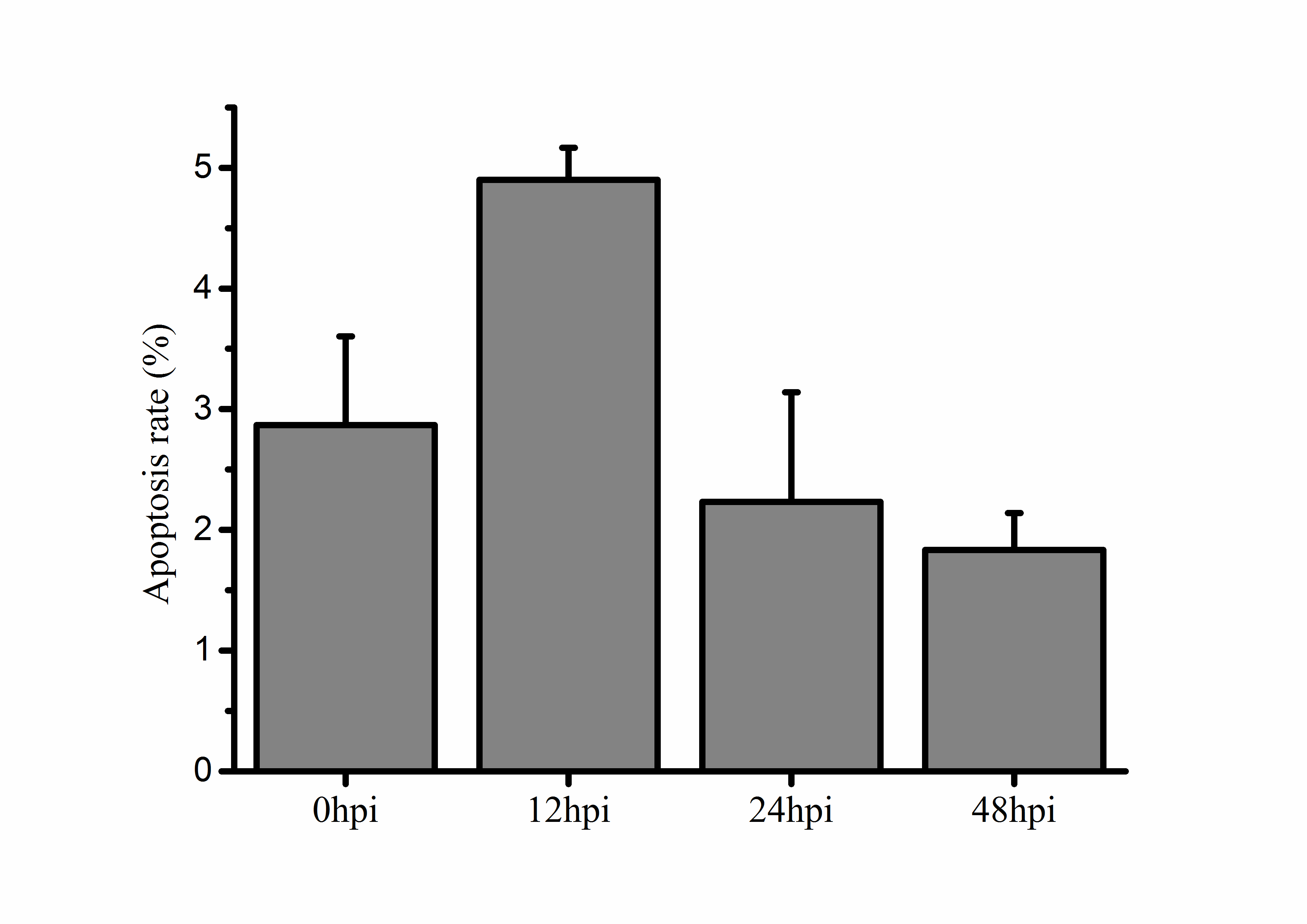


**Supplementary Figure 4.** The apoptosis rate of haemocytes at 0 h, 12 h, 24 h and 48 h post-*V*. *parahaemolyticus* injection.
